# ChatGPT Surpasses 1000 Publications on PubMed: Envisioning the Road Ahead

**DOI:** 10.7759/cureus.44769

**Published:** 2023-09-06

**Authors:** Mohamad-Hani Temsah, Ibraheem Altamimi, Amr Jamal, Khalid Alhasan, Ayman Al-Eyadhy

**Affiliations:** 1 Pediatric Intensive Care Unit, King Saud University, Riyadh, SAU; 2 College of Medicine, King Saud University, Riyadh, SAU; 3 Family and Community Medicine, King Saud University, Riyadh, SAU; 4 Pediatric Nephrology, King Saud University, Riyadh, SAU; 5 Pediatrics, King Saud University, Riyadh, SAU; 6 Pediatric Intensive Care Unit, King Saud University Medical City, Riyadh, SAU

**Keywords:** published in pubmed journal, randomized controlled trial (rct), ai chatbot, ethical implications, medical education, telemedicine, artificial intelligence (ai), llms in healthcare, pubmed, chatgpt

## Abstract

The exponential growth of ChatGPT in medical literature, amassing over 1000 PubMed citations by August 2023, underscores a pivotal juncture in the convergence of artificial intelligence (AI) and healthcare. This remarkable rise not only showcases its potential to revolutionize medical academia but also indicates its impending influence on patient care and healthcare systems. Notwithstanding this enthusiasm, one-third of these citations are editorials or commentaries, stressing a gap in empirical research. Alongside its potential, there are concerns about ChatGPT becoming a "Weapon of Mass Deception" and the need for rigorous evaluations to counter inaccuracies. The World Association of Medical Editors has released guidelines emphasizing that AI tools should not be manuscript co-authors and advocates for clear disclosures in AI-assisted academic works. Interestingly, ChatGPT achieved its citation milestone within nine months, compared to Google's 14 years. As Large Language Models (LLMs), like ChatGPT, become more integral in healthcare, issues surrounding data protection, patient privacy, and ethical implications gain prominence. As the future of LLM research unfolds, key areas of interest include its efficacy in clinical settings, its role in telemedicine, and its potential in medical education. The journey ahead necessitates a harmonious partnership between the medical community and AI developers, emphasizing both technological advancements and ethical considerations.

## Editorial

**Figure 1:**
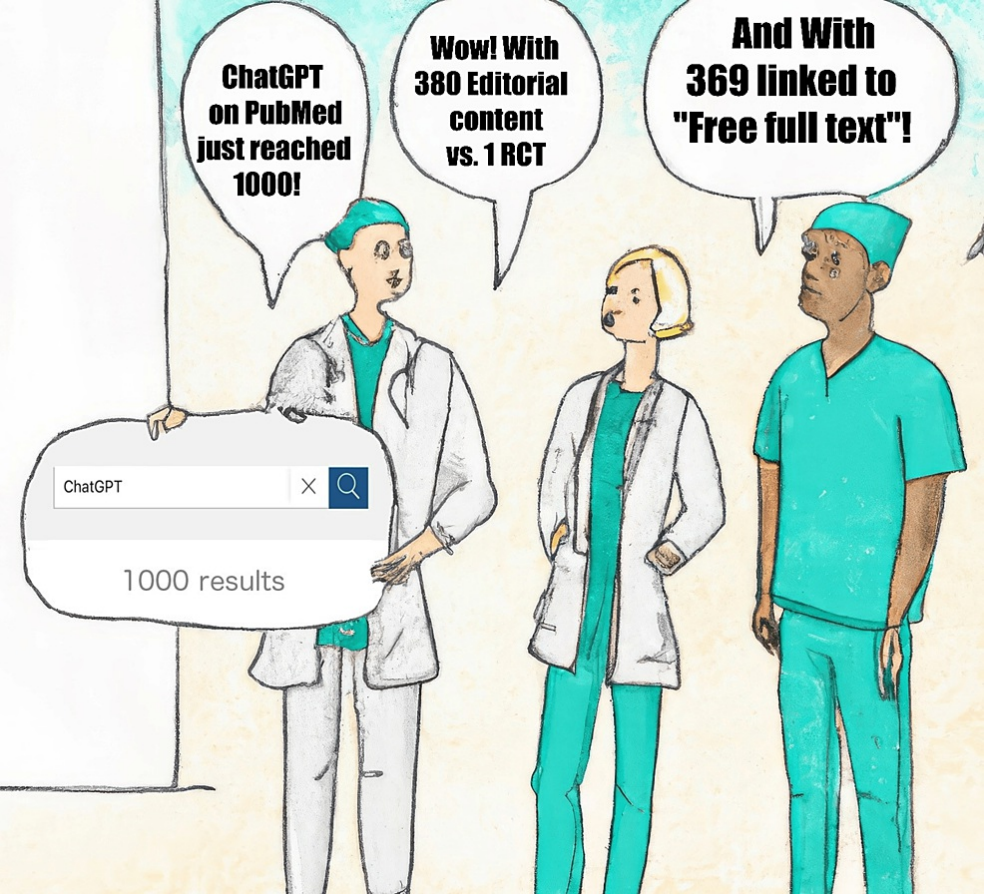
Example of Human-AI-Human interaction Imaginary figure that was initially drafted using AI-based OpenAI’s DALLE-2 software, with the prompt: “Draw 3 healthcare professionals in the hospital talking together, with 3 empty chat boxes/balloons, digital art". The drawing was then edited by the authors; with a merging of the number of papers’ type Editorial content (Editorial, Comment, and Letter), free open access, and Randomized Controlled Trial (RCT) filters applied on the PubMed website, using “ChatGPT” as the search keyword; at the time the search output reached 1000 on August 10th, 2023.

The rapid rise of ChatGPT (Chat Generative Pre-trained Transformer) in medical literature, with over 1000 PubMed citations by August 2023 (Figure 1), signals a critical moment in blending artificial intelligence (AI) and healthcare. This “new” large language model (LLM) illustrated vast prospects for transforming medical academia and, potentially, healthcare practices. Its influence may soon extend beyond research to practical applications, reshaping patient care and healthcare systems. However, of these PubMed citations, a notable one-third are editorials or commentaries, highlighting the need for more empirical studies.

The “buzz” around ChatGPT's healthcare pros and cons has been undeniable since its debut, with debate on the potential to affect medical development and advocacy for more vigilance [1]. OpenAI's ChatGPT showed diverse medical applications from its early days, that flourished in the medical literature [2]. Yet, alongside the enthusiasm are reservations hindering its optimal adoption in medicine. OpenAI promotes ChatGPT's potential but also cautions about potential inaccuracies with a disclaimer on its website. This highlights the importance of rigorous expert evaluation and continuous refinement of the AI model.

Recent guidelines by the World Association of Medical Editors (WAME) emphasize caution in ChatGPT's role in scientific literature authorship [3]. The consensus is clear: AI chatbots, ethically and legally, should not be manuscript co-authors. The need for standardized reporting and AI-tool usage checklists in medical research is pressing. WAME urges detailed disclosures about the AI tool used, including its name, version, and specific prompts. Such transparency is fundamental to ensure credibility and trustworthiness in AI-assisted academic writing. On the other hand, we suggest that ChatGPT and other LLMs have the potential to serve as personal assistants for journal editors and reviewers. By automating some repetitive tasks, these AI tools could optimize and facilitate their workflow, allowing for a possibly more efficient review process, though more research and guidance are needed in this regard.

OpenAI introduced ChatGPT on November 30, 2022, whereas Google's inaugural version was unveiled in August 1996 via Stanford's website. Notably, by 2001, Google had garnered a mere three citations on PubMed, but it achieved the benchmark of 1000 papers by early 2010. In contrast, ChatGPT, having its first PubMed paper in December 2022, reached its thousand milestone in a mere nine months. The integration of ChatGPT and similar innovative technologies into healthcare echoes the diffusion of innovations theory; starting with pioneers and early adopters, subsequently gaining momentum as its utility becomes apparent [4]. As the theory suggests, after the initial wave of early adopters -those pioneering healthcare professionals and entities quick to grasp the potential of new technologies - the early majority steps in. This group pragmatically adopts the innovation after witnessing its tangible benefits and potentials, driving the usage to a critical mass. Following them, the late majority adopts the technology as it solidifies its position as the “new norm”. Eventually, the laggards, often the most cautious, integrate it, driven by its ubiquity and the industry's necessity. The degree and pace of adoption are influenced by perceived advantages, accompanying costs, regulatory frameworks, and alignment with current healthcare systems. Exploring these dynamics is central to crafting strategies that promote proper LLMs’ integration into healthcare and managing potential obstacles.

As ChatGPT evolves, its healthcare role expands, highlighting potential clinical and research roles [5]. It can soon analyze updated medical literature, providing insights and research guidance. Yet, concerns arise about reliability and human oversight. Notably, while most ChatGPT papers on PubMed have full-text links, only one-third are open-access, revealing a data-sharing opportunity. Therefore, publishers and funding bodies could facilitate more open-access publications, as that could empower scientists and researchers with valuable and timely information in such rapidly evolving fields. With just one randomized control trial (RCT) on ChatGPT in PubMed, there is a need for more comprehensive research and scholarly efforts in this domain. This emphasizes the importance of diversifying the types of studies conducted on ChatGPT and other LLMs, to ensure a holistic evaluation of their capabilities and limitations.

The rapid growth of ChatGPT and other LLMs in medical literature also recalls to the forefront the question of data protection and patients’ privacy. As AI becomes more integrated into healthcare systems, ensuring the confidentiality of patient data becomes paramount. Moreover, the potential for AI to assist in diagnostic or clinical management processes necessitates rigorous validation to prevent misdiagnoses or oversight. The ethical implications of LLMs’ role in patient care, especially in terms of data privacy or AI-generated bias, cannot be overstated.

Envisioning the road ahead in future LLM research, vital explorations include evaluating ChatGPT's algorithms in clinical contexts, its adaptability across diverse patients, and its accuracy in complex diseases. Equally important is understanding its synergy with other medical technology and ensuring ethical implementation. Another key area of exploration is the potential for AI to transform telemedicine. With the rise of remote consultations, especially with the COVID-19 pandemic, AI chatbots like ChatGPT could play a substantial role in enhancing patient-doctor interactions, making them more efficient and informed. Their potential to bridge geographical barriers and provide instant medical insights could optimize telehealth at a global level.

The exploration of LLM's role in medical education is of high importance and should be conducted concurrently, if not preemptively, with healthcare industry advancements and technical developments. As we stand on the crossover of a transformative era in medical education, the potential benefits of LLMs are undeniable. They could substantially facilitate learning, offering students unparalleled access to vast repositories of knowledge and cutting-innovative insights. However, this optimism is tempered by valid concerns: Could such tools inadvertently foster an over-reliance, leading to student dependency? It is essential that research efforts strike a balance, harnessing the positive potential of LLMs while also addressing and mitigating potential pitfalls. Only through such a weighed approach can we truly steer the integration of LLMs in a way that maximizes their utility and acceptance within the medical education community. In preparing our future healthcare professionals, the challenge lies in ensuring that while students leverage the advantages of LLMs, they also develop critical thinking and problem-solving skills independently.

As ChatGPT literature reaches its initial milestone on PubMed, it becomes crucial to broaden our scope and consider other global literature and scientific research repositories. This encompasses medical databases, such as Europe PMC or the Latin America and the Caribbean Literature on Health Sciences (LILACS) databases. Additionally, platforms that cater to AI scientists and software engineers need to be explored, such as the Institute of Electrical and Electronics Engineers (IEEE) Xplore digital library. Embracing a diverse, multinational, and multilingual approach is essential in this context. The expanding need for collaboration between healthcare professionals and computer experts highlights this perspective.

Evidently, what we have witnessed represents merely the initial strides in an ample journey of continuous learning, adaptation, and partnership between the medical communities and AI developers. This collaboration should aim to optimize patient care and advance medical research. As the field evolves, the future demands a balanced approach to technological progress and ethical management of LLMs in medicine. Adapting AI chatbots to align with medical community needs and standards is crucial. This ensures these technologies become assets rather than liabilities.
